# Whole Genome Sequencing of SARS-CoV-2 in Cats and Dogs in South Korea in 2021

**DOI:** 10.3390/vetsci10010006

**Published:** 2022-12-23

**Authors:** Yeun-Kyung Shin, Oh-Kyu Kwon, Jinhwa Heo, Jinju Nah, Hae-Eun Kang, Yunhee Kang, In Jun Song, Ho-Kyung Sung

**Affiliations:** 1 Foreign Animal Disease Division, Animal and Plant Quarantine Agency, Gimcheon 39660, Republic of Korea; 2 Animal Health Center, Research Institute of Public Health and Environment, Seoul Metropolitan Government, Gwacheon 13818, Republic of Korea; 3 Animal Welfare Division, Parks and Recreation Bureau, Seoul Metropolitan Government, Seoul 04524, Republic of Korea

**Keywords:** SARS-CoV-2, animals, variants, dogs, cats, genomic, surveillance

## Abstract

**Simple Summary:**

Forty SARS-CoV-2-positive swab samples from 40 animals owned by confirmed SARS-CoV-2-infected owners in Korea in 2021 were analyzed using next-generation sequencing (NGS) to identify the genomic lineage of the viruses. Among the 40 animal samples, eight Pango lineages (B.1.1.7) (Alpha variant), B.1.429 (Epsilon variant), B.1.470, B.1.497, B.1.619.1, B.1.620, AY.69 (Delta variant), and AY.122.5 (Delta variant) were identified. This study provides the first reported cases of six lineages (B.1.470, B.1.497, B.1.620, B.1.619.1, AY.69 (Delta variant), and AY.122.5 (Delta variant)) in cats and dogs. Our results emphasize the importance of monitoring SARS-CoV-2 in pets because they are dynamic hosts of variant Pango lineages of SARS-CoV-2.

**Abstract:**

SARS-CoV-2 infections have caused unprecedented damage worldwide by affecting humans and various animals. The first reported animal infection was observed in a pet dog in Hong Kong in March 2020. 36 countries reported 692 SARS-CoV-2 infections in 25 different animal species by 31 August 2022. Most outbreaks were caused by contact with SARS-CoV-2 infected humans. In South Korea, the first SARS-CoV-2 infection in an animal was reported in a cat in February 2021. As of 31 December 2021, 74 dogs and 42 cats have been confirmed to have SARS-CoV-2 in South Korea. Here, we identified various SARS-CoV-2 genomic lineages in SARS-CoV-2 confirmed cats and dogs. Among the 40 animal samples sequenced for lineage identification, a total of eight Pango lineages (B.1.1.7 (Alpha variant), B.1.429 (Epsilon variant), B.1.470, B.1.497, B.1.619.1, B.1.620, AY.69 (Delta variant), and AY.122.5 (Delta variant)) were identified. The dominant lineages were AY.69 (Delta variant; 37.5%), B.1.497 (35.0%), and B.1.619.1 (12.5%). This study provides the first reported cases of six lineages (B.1.470, B.1.497, B.1.620, B.1.619.1, AY.69 (Delta variant)), and AY.122.5 (Delta variant) in cats and dogs. Our results emphasize the importance of monitoring SARS-CoV-2 in pets because they are dynamic hosts of variant Pango lineages of SARS-CoV-2.

## 1. Introduction

Coronavirus disease 2019 (COVID-19) has caused unprecedented global damage by affecting not only humans, but also various animals. COVID-19 was first reported in humans in Wuhan, China, in December 2019, and was caused by SARS-CoV-2 [1]. As of October 2022, about 630 million people have been infected with SARS-CoV-2, and about 6.5 million of them have been confirmed dead [2]. SARS-CoV-2 is a positive-stranded RNA virus belonging to the family *Coronaviridae* of the order *Nidovirales* [3]. Coronaviruses are known to have a virus mutation rate about 10 times lower than that of influenza viruses because they have an exonuclease enzyme that lowers the replication error rate [4]; however, similar to many other RNA viruses, the SARS-CoV-2 genome has been constantly mutating to adapt to the host immune system since its emergence [5,6]. The evolution of the SARS-CoV-2 gene has resulted in tens of thousands of mutations in the SARS-CoV-2 genome, which has caused continuous public health difficulties such as vaccines, treatments, and application of diagnostic technologies [7]. The World Health Organization (WHO) has classified SARS-CoV-2 with mutations of high impact as variants of concern (VOC), and to date, five major VOC have been detected: the Alpha (B.1.1.7), Beta (N.1.351), Gamma (P.1), Delta (B.1.617.2), and Omicron (B.1.1.529) variants [2].

SARS-CoV-2 spillover from humans to domestic cats, dogs, and other animals has been reported globally [8]. The first reported animal infection was observed in Hong Kong in March 2020 when a pet dog was infected with SARS-CoV-2 [9]. In total, 36 countries had reported 692 SARS-CoV-2 infections in 25 different animal species (cat, dog, mink, otter, pet ferret, lion, tiger, puma, snow leopard, gorilla, white-tailed deer, fishing cat, binturong, South American coati, spotted hyena, Eurasian lynx, Canada lynx, hippopotamus, hamster, mule deer, giant anteater, west Indian manatee, blacktailed marmoset, common squirrel monkey, and mandrill) as of 31 August 2022 [8]. The actual number of animal infections was expected to be higher than 692 as the number of SARS-CoV-2 sequences uploaded in Global Initiative for Sharing All Influenza Data (GISAID) was >2400 as of 8 December 2022 [10]. Most of these infections are believed to be spillovers from humans, except for a few cases such as mink-to-human infections in European countries [11,12,13] and hamster-to-human infections in Hong Kong [14,15,16].

In South Korea, the first case of SARS-CoV-2 infection in animals was reported on 29 January 2021, in a domestic cat in Gyeongnam province, residing in a religious facility, where multiple individuals (120 residents and visitors) were confirmed to have SARS-CoV-2 infection [17]. The virus lineage was B.1.497 (Pango v.2.3.2), and the virus genomes found in the owner and the cat were identical. Based on an epidemiological investigation of the facility, it was believed that the owner transmitted the virus to cat. This event raised public concern, and guidelines for the management of COVID-19 in pet cats and dogs were established by the Ministry of Agriculture, Food and Rural Affairs (MAFRA) in South Korea in February 2021. The guidelines specified that pets owned by COVID-19 confirmed patients should be tested in provincial government veterinary laboratories if the animals had been in contact with the confirmed patients and showed suspected clinical symptoms of COVID-19. Between February and December 2021, 116 animals (42 cats and 74 dogs) were confirmed to be infected with SARS-CoV-2 (MAFRA internal document, 2022). In this study, 40 SARS-CoV-2-positive swab samples from 40 animals were analyzed using next-generation sequencing (NGS) to identify the genomic lineage of the viruses. Among the 40 animal samples, a total of eight Pango lineages (B.1.1.7 (Alpha variant), B.1.429 (Epsilon variant), B.1.470, B.1.497, B.1.619.1, B.1.620, AY.69 (Delta variant), and AY.122.5 (Delta variant)) were identified. This study provides the first reported cases of six lineages (B.1.470, B.1.497, B.1.620, B.1.619.1, AY.69 (Delta variant), and AY.122.5 (Delta variant)) in cats and dogs. Our results emphasize the importance of monitoring SARS-CoV-2 in pets because they are dynamic hosts of variant Pango lineages of SARS-CoV-2.

## 2. Materials and Methods

### 2.1. Sample Collection and Detection of SARS-CoV-2

In total, 110 SARS-CoV-2 positive domestic animal (41 cats and 69 dogs) sample sets (oropharyngeal and rectal swabs, as well as nasopharyngeal swabs) were collected in seven South Korean municipalities (Seoul, Daegu, Gwangju, Sejong, Gyeonggi-do, Gyeongnam-do, and Jeonbuk-do) between February and December 2021 and sent to the Animal and Plant Quarantine Agency (APQA) for whole genome sequencing (Supplementary Table S1). All owners of the sampled animals were confirmed to be SARS-CoV-2 positive by the Ministry of Health, and the animal samples were collected and tested the day of, or one to two days after confirmation. To identify the presence of the SARS-CoV-2 genome, commercially available real-time RT-PCR assays (Kogene Biotech, Seoul, South Korea) targeting the RNA-dependent RNA polymerase (RDRP) gene and envelope (E) protein gene were performed according to the manufacturer’s instructions. Briefly, viral RNA was extracted using the Maxwell^®^ RSC Total Nucleic Acid kit (Promega, Madison, WI, USA) according to the manufacturer’s instructions. Real-time RT-PCR was performed using a Bio-Rad thermocycler (Bio-Rad, Hercules, CA, USA). Animal samples with a cycle threshold (Ct) value of 20–30 were then subjected to NGS of the whole genome of the virus.

### 2.2. Next-Generation Sequencing and Assembly

RNA was converted to cDNA using the PrimeScript RT reagent kit (Takara Bio Europe SAS, Saint-Germain-en Laye, France) with a combination of oligo-dT and random hexamer methods, following the manufacturer’s protocol. cDNA was used for viral DNA enrichment using the Qiagen SARS-CoV-2 Primer Panel (Qiagen, Hilden, Germany). The amplified PCR products were used for sequencing-ready library preparation using an Illumina DNA LibraryPrep kit (Illumina, San Diego, CA, USA). The prepared libraries were loaded onto the Illumina Mini-Seq platform and a 150 bp paired-end sequencing kit (300 cycles).

Raw reads were trimmed using the Bbduk software (v38.96) [18]. Trimmed reads were then aligned to the SARS-CoV-2 Wuhan-Hu-1 reference sequence (NC_045512.2) using the BWA software (v1.9.0) [19]. Only uniquely mapped reads extracted using SAMtools (v1.15.1) were considered for downstream analyses [20]. The reads mapped to the genes were counted using gff2bed (v24.3.9) [21]. The aligned reads were sorted, variant calling was performed using GATK variant caller v4.6.2.1 [22], and a consensus sequence was generated from each sample using BCFTools v0.1.16. with default parameters [23].

### 2.3. Genotyping and Phylogenetic Analysis

Lineage classification was performed using the Pangolin tool (v.4.1.3) based on the Pango Dynamic Nomenclature System [24,25]. Phylogenetic analysis of the whole-genome sequencing data of the samples was performed by multiple sequence alignment of the sequences with the complete genomes available in the GISAID database. A total of 26,373 complete genome sequences of SARS-CoV-2 strains reported in South Korea were retrieved from the GISAID database on 16 October 2022. Among the 26,373 complete genome sequences, 800 were selected by blasting each animal sample sequence and choosing the 20 sequences showing the highest homology with each sample sequence. After removing redundant sequences, 421 were selected (Supplementary Table S3). The 461 (421 and 40) sequences were aligned using MAFFT v7.508 and a phylogenetic tree was constructed using IQ-TREE v.2.2.0 to apply the GTR + F model of nucleotide substitution with default heuristic search options and bootstrapping with 1000 replicates [26,27].

## 3. Results

### 3.1. SARS-CoV-2 Whole Genome Sequencing in Cats and Dogs

All samples collected from SARS-CoV-2 confirmed pets of SARS-CoV-2 confirmed owners were tested for SARS-CoV-2 genomic surveillance. Forty samples (25 cats and 15 dogs) showed Ct values of approximately 20 to 30 in real-time RT-PCR, and they were subjected to whole genome sequencing using Illumina. The run produced an average of 3,825,036 reads and the average coverage was 2126.8 with a read depth of 20x covering 97.0% of the full-length genome of the reference genome (Supplementary Table S2). Forty full-genome sequences were generated, with an average genome size of 29,891 bp.

### 3.2. Molecular and Phylogenetic Characterization of Viral Genomes

The resulting SARS-CoV-2 genomes were compared with the reference NC_045512.2 (Wuhan-Hu-1) for genotyping. One Alpha, one Epsilon, 17 Delta, and 21 other variants were identified. All identified lineages based on the Pango Dynamic Nomenclature System are presented in Table 1. Five Pango lineages (B.1.497, B.1.619.1, B.1.620, AY.69 (Delta variant), and AY.122.5 (Delta variant)) were identified in dogs, and seven Pango lineages (B.1.1.7 (Alpha variant), B.1.429 (Epsilon variant), B.470, B.1.497, B.1.619.1, AY.69 (Delta variant), and AY.122.5 (Delta variant)) were identified in cats. Four Pango lineages (B.1.497, B.1.619.1, AY.69 (Delta variant), and AY.122.5 (Delta variant)) were identified in both species. The most dominant Pango lineages were AY.69 (Delta variant) (37.5%), B.1.497 (35.0%), and B.1.619.1 (12.5%).

**Figure 1:**
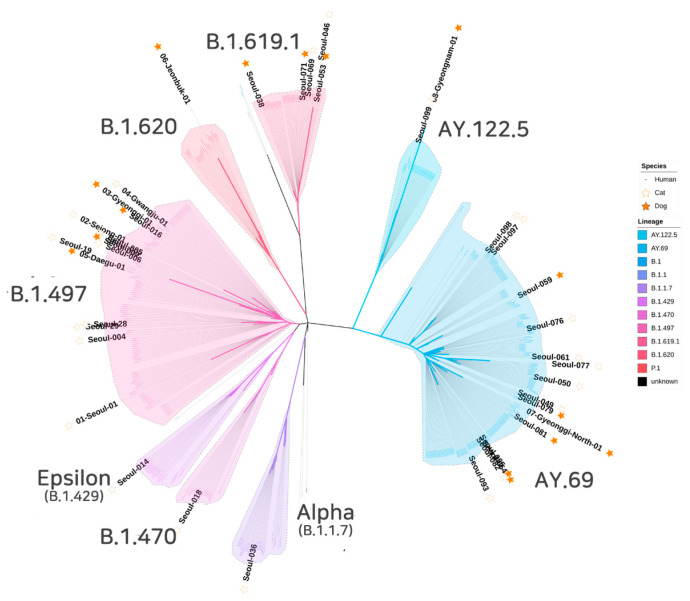
Phylogenetic tree analysis of SARS-CoV-2 whole genome sequences. This tree includes 40 SARS-CoV-2 whole genome sequences obtained in this study (25 dogs and 15 cats) and 421 SARS-CoV-2 complete genome sequences in GISAID from humans in South Korea. The analysis used ML with the GTR and F model.

Phylogenetic analysis was performed by comparing the 40 complete genomes of SARS-CoV-2 from cats and dogs in this study and SARS-CoV-2 human genomes from South Korea available in the GISAID database. The 421 viral genome sequences were used to infer a maximum likelihood tree using a dataset comprising all SARS-CoV-2 genomes identified in South Korea. All eight lineages identified in South Korean cats or dogs were grouped in the lineage branches of South Korean human SARS-CoV-2 isolates (Figure 1).

The 40 full genomes generated in this study were submitted to GISAID (Accession ID. EPI_ISL_15727682~ EPI_ISL_15727702, EPI_ISL_15775580~ EPI_ISL_15775595, EPI_ISL_15788906, EPI_ISL_15793141, and EPI_ISL_15793142, Table 2).

## 4. Discussion

To the best of our knowledge, our study is the first report of SARS-CoV-2 whole genome sequencing of cats and dogs in South Korea. A total of 391 pet samples were tested for SARS-CoV-2, 116 of which were positive (29.7 %), and 110 positive samples were finally submitted to APQA for genome sequencing.

In this study, 40 animal samples from 110 SARS-CoV-2 positive pet cats and dogs of by SARS-CoV-2 confirmed owners were sequenced using Illumina NGS. Most animal samples (102, 92.7 %) were from Seoul, where 18.3% of the total population of South Korea reside and the second biggest pets population (18.4%) out of 2,782,811 pet cats and dogs is registered [28].

In total, eight Pango lineages (B.1.1.7 (Alpha variant), B.1.429 (Epsilon variant), B.1.470, B.1.497, B.1.620, B.1.619.1, AY.69 (Delta variant), and AY.122.5 (Delta variant)) were identified in this study. Dominant lineages were AY.69 (Delta variant; 37.5%), B.1.497 (35.0%), and B.1.619.1 (12.5%). In cats, B.1.497 was the most detected lineage (40.0%), followed by AY.69 (Delta variant; 36.0%). In dogs, AY.69 (Delta variant) was the most detected lineage (40.0%), followed by B.1.497 (26.7%).

Since the first human SARS-CoV-2 case in January 2020, 630,783 confirmed human cases, with 5563 deaths, have been reported in South Korea, as of December 2021 [29]. All eight SARS-CoV-2 lineages detected in cats and dogs in this study were also found in humans in South Korea. The Pango lineage B.1.1.7 (Alpha variant) was first detected in the obtained human samples in December 2020, and was last detected in September 2021 in South Korea [10]. The total number of human B.1.1.7 sequences uploaded in GISAID is 862. This lineage has been detected in at least 41 countries, most commonly in the United Kingdom (24.0%), the USA (20.0%), Germany (9%), Sweden (6.0%), and Denmark (6.0%) [30]. B.1.1.7 (Alpha variant) was reported in a cat and dog in the USA in February 2021 [31]. It has also been detected in cats and dogs in Argentina, Bosnia and Herzegovina, France, Italy, Latvia, and Thailand in 2021 [10]. In this study, B.1.1.7 (Alpha variant) was detected in a cat in Seoul in June 2021, approximately six months after the first human B.1.1.7 case in South Korea.

The B.1.429 lineage (Epsilon variant) was first detected in South Korea in December 2020, and was last detected in samples collected in June 2021 [10]. A total of 107 human B.1.429 sequences have been deposited in GISAID. This lineage has been detected in at least 45 countries, most commonly in the USA (97.0%), Canada (2.0%), and Mexico (1.10%) [30]. B.1.429 (Epsilon variant) was reported in cats in the USA in February 2021 [10]. In this study, B.1.429 (Epsilon variant) was detected in a cat in Seoul in March 2021, approximately three months after the first human B.1.429 (Epsilon variant) case.

The B.1.470 lineage was first detected in South Korea in December 2020 and was last detected in the samples collected in May 2021 [10]. A total of 25 sequences from Korea are uploaded in GISAID. This lineage has been detected in at least 29 countries, most commonly in Indonesia (62.0%), Malaysia (5.0%), South Korea (3.0%), Singapore (3.0%), and Japan (2.0%) [30]. There have been no official reports of confirmed cases of this lineage in cats and dogs. In this study, B.1.470 was detected in a cat in Seoul in April 2021, approximately four months after the first human B.1.470 case.

The B.1.497 lineage was first detected in South Korea in March 2020 and was a dominant subgroup until April 2021 [32]. This lineage was dynamically associated with visitors to clubs and bars in Itaewon, Seoul, from April 29 to 6 May 2020, and caused one of the major local and long-term epidemics in South Korea [33]. A total of 3180 sequences in Korea have been uploaded in GISAID. The B.1.497 lineage has been detected in at least 11 countries, most commonly in South Korea (98.6%), the USA (0.65%), and Japan (0.06%) [30]. There have been no official reports of confirmed cases of this lineage in cats and dogs. In this study, B.1.497 was detected in a cat in Seoul in February 2021, approximately seven months after the first human B.1.497 case. In total, this study detected this lineage in 14 animals (10 cats and four dogs).

The earliest sequences of B.1.619.1 and B.1.620 were reported in South Korea in February and March 2021, respectively [10]. KCDA carried out whole genome sequencing when the number of infections by B.1.619 and B.1.620 increased sharply (April–July 2021), and the prevalence rates increased rapidly to 67% in June 2021 and decreased when the Delta variant emerged in July [32]. B.1.619 and B.1.620 were identified in imported cases in South Korea in 2021, and B.1.619.1 has been reclassified as it has an additional mutation in ORF1a (K3929R) [31,32]. A total of 1595 and 412 B.1.619.1 and B.1.620 sequences in Korea, respectively, have been uploaded in GISAID. Both B.1.619.1 and B.1.620 were last detected in samples collected in September 2021. B.1.619.1 was confirmed in at least 13 countries, most commonly in South Korea (100.0%) and Belgium (1.0%) [30]. B.1.620 was confirmed in at least 37 countries, most commonly in South Korea (32.0%), Lithuania (15.0%), France (11.0%), Republic of the Congo (6.0%), and Italy (5.0%) [30]. There have been no official reports of confirmed cases of B.1.619.1 and B.1.620 in cats and dogs. In this study, B.1.619.1 was detected in a dog in Seoul in June 2021, four months after the first human B.1.619.1 case, as well as in two additional dogs and two cats. B.1.620 was detected in a dog in Jeonbuk-do in May 2021, three months after the first human B.1.620 case.

The AY.69 lineage (Delta variant, alias of B.1.617.2.69) was first reported in human samples collected in South Korea in May 2021 [10], and became the dominant lineage (56.7%) together with AY.122.5 (19.7%) from April 2020 to January 2022 [34]. A total of 11,348 AY.69 sequences have been uploaded in GISAD: AY.69 was last detected in January 2022 [10]. This lineage has been detected in at least 16 countries, most commonly in South Korea (100.0%) [30]. There have been no official reports of confirmed cases of this lineage in cats and dogs. In this study, AY.69 (Delta variant) was detected in a cat in Seoul in July 2021, two months after the first human AY.69 (Delta variant) case. Since then, it has been detected in eight cats and six dogs.

The AY.122.5 lineage (Delta variant, alias of B.1.617.2.122.5) was first detected in South Korea in August 2021 [10]. It became dominant among Delta variants in South Korea in December 2021 and was the second most detected lineage (19.5%) in January 2022, while Omicron BA.1.1. was the most detected (53.2%) [34]. AY.122.5 was last detected in February 2022, and a total of 4141 sequences from Korea have been uploaded [10]. This lineage has been detected in at least eight countries, most commonly in South Korea (99.0%) and the USA (15.0%) [30]. There have been no official reports of confirmed cases of this lineage in cats and dogs. In this study, AY.122.5 (Delta variant) was detected in a cat in Seoul in October and in a dog in Gyeongnam-do in December 2021, two to four months after the first human AY.122.5 (Delta variant) case.

Korea Disease Control and Prevention Agency (KDCA) performed genomic surveillance on 17.6% human samples submitted for SARS-CoV-2 diagnosis from April 2021 to January 2022, which is a similar samples collection period to that of the current study [34]. The most dominant variant in humans was the Alpha variant (13.7–24.4%) from April to June, and the Delta variant (59.6–96.1%) from July to December in 2021. In detail, AY.69 and AY.122.5 were the dominant Delta variant sub-lineages in humans during the fourth wave of COVID-19 (July–December 2021), when the Delta variant was the major variant in South Korea. In our study, AY.69 and AY.122.5 were also the most dominant lineages (73.9%, 17 out of 20) in cats and dogs during the same period. In another study carried out by KDCA, B.1.497 was the most dominant Pango lineage from April 2020 to April 2021 [32]. In this study, 12 (85.7%) out of 14 samples collected during February-April 2021 are B.1.497 Pango lineage.

The eight Pango lineages identified in cats and dogs were all grouped into the same South Korean human SARS-CoV-2 lineage branches. The delays between the first human and animal cases were between 2 and 9 months, and all animal cases were confirmed after the same Pango lineages were identified in humans. Therefore, the SARS-CoV-2 viruses that circulated in South Korea are believed to have shared hosts, and the results provide additional evidence for the theory that most animal infections of SARS-CoV-2, especially in cats and dogs, are the spillover from humans. We have confirmed that most of the defining mutations of each lineage exist in each genome. Further study is needed to identify any specific mutations that show species-specific characteristics.

Among the eight lineages detected in cats or dogs in this study, four lineages (B.1.497, B.1.619.1, AY.69 (Delta variant), and AY.122.5 (Delta variant)) are considered South Korean lineages [30], because they have been reported for the first time in South Korea, and most of the genome sequences deposited in GISAID are from South Korea (98.6% of B.1.497, 100% of B.1.619.1, 99.0% of AY.69 (Delta variant), and 100% of AY.122.5 (Delta variant). These four lineages were also the four most dominant lineages in the animals in this study (AY.69, 37.5%, B.1.497, 35%, B.1.619.1, 12.5%, and AY.122.5, 5%).

According to GISAID, as of 16 October 2022, 71 and 46 SARS-CoV-2 Pango lineages have been detected in cats and dogs, respectively. However, B.1.497, B.1.619.1, AY.69 (Delta variant), and AY.122.5 (Delta variant), which are South Korean lineages, and B.1.470 and B.1.620 have, to date, not been reported in cats and dogs. To the best of our knowledge, this is the first report of a molecular epidemiological study of SARS-CoV-2 infection in cats and dogs living under natural conditions in South Korea, and of the infection of SARS-CoV-2 lineages B.1.470, B.1.497, B.1.619.1, B.1.620, AY.69 (Delta variant), and AY.122.5 (Delta variant) in cats and dogs globally. The data obtained in this study emphasize the need for further epidemiological studies and consistent surveillance of SARS-CoV-2 in animals. Our study highlights the importance of genomic surveillance to monitor the emergence of new viral lineages in different species.

## 5. Conclusions

We identified various SARS-CoV-2 genomic lineages in cats and dogs. Among 40 animal samples sequenced for lineage identification of SARS-CoV-2, eight Pango lineages (B.1.1.7 (Alpha variant), B.1.429 (Epsilon variant), B.1.470, B.1.497, B.1.619.1, B.1.620, AY.69 (Delta variant), and AY.122.5 (Delta variant)) were identified, and dominant lineages were AY.69 (Delta variant), B.1.497, and B.1.619.1 in both cats and dogs. All eight lineages were confirmed to have simultaneously occurred in humans in South Korea. Importantly, six lineages (B.1.470, B.1.497, B.1.620, B.1.619.1, AY.69 (Delta variant), and AY.122.5 (Delta variant)) were first reported in cats and dogs in this study. Our results validate that monitoring SARS-CoV-2 in pets is important and that cats and dogs are dynamic hosts of SARS-CoV-2.

## Figures and Tables

**Table 1:** Number of sequences of variant strains of SARS-CoV-2 in 40 animal samples.

Pango Lineage		Cat	(%) *	Dog	(%) *	Total	(%) *
B.1.497		10	40.0	4	26.7	14	35.0
B.1.429	(Epsilon variant)	1	4.0	-	0.0	1	2.5
B.1.470		1	4.0	-	0.0	1	2.5
B.1.620		-	0.0	1	6.7	1	2.5
B.1.1.7	(Alpha variant)	1	4.0	-	0.0	1	2.5
B.1.619.1		2	8.0	3	20.0	5	12.5
AY.69	(Delta variant)	9	36.0	6	40.0	15	37.5
AY.122.5	(Delta variant)	1	4.0	1	6.7	2	5.0
Total		25		15		40	

* The frequency of the lineage among positive samples in cats or dogs.

**Table 2:** Pangolin lineages of SARS-CoV-2 in 40 animal samples.

No.	Species	Strain	Sampling Date	Pango Lineage	Nextstrain Clade †	GISAID Clade ‡	WHO Label	GISAID Accession ID.
1	cat	01-Seoul-01	2021-02-13	B.1.497	20C	GH	–	EPI_ISL_15775580
2	cat	02-Sejong-01	2021-02-16	B.1.497	20C	GH	–	EPI_ISL_15775581
3	dog	Seoul-002	2021-02-16	B.1.497	20C	GH	–	EPI_ISL_15775592
4	dog	03-Gyeonggi-01	2021-02-19	B.1.497	20C	GH	–	EPI_ISL_15788906
5	cat	04-Gwangju-01	2021-03-05	B.1.497	20C	GH	–	EPI_ISL_15793141
6	cat	Seoul-004	2021-03-08	B.1.497	20C	GH	–	EPI_ISL_15775582
7	cat	Seoul-005	2021-03-09	B.1.497	20C	GH	–	EPI_ISL_15727682
8	cat	Seoul-006	2021-03-09	B.1.497	20C	GH	–	EPI_ISL_15727683
9	cat	Seoul-007	2021-03-09	B.1.497	20C	GH	–	EPI_ISL_15727684
10	cat	Seoul-014	2021-03-26	B.1.429	21C (Epsilon)	GH	Epsilon Variant	EPI_ISL_15727685
11	dog	05-Daegu-01	2021-03-30	B.1.497	20C	GH	–	EPI_ISL_15727696
12	dog	Seoul-016	2021-04-06	B.1.497	20C	GH	–	EPI_ISL_15775593
13	cat	Seoul-018	2021-04-11	B.1.470	20A	GH	–	EPI_ISL_15727686
14	cat	Seoul-19	2021-04-13	B.1.497	20C	GH	–	EPI_ISL_15727687
15	cat	Seoul-28	2021-05-04	B.1.497	20C	GH	–	EPI_ISL_15727688
16	cat	Seoul-29	2021-05-04	B.1.497	20C	GH	–	EPI_ISL_15727689
17	dog	06-Jeonbuk-01	2021-05-28	B.1.620	20A	G	–	EPI_ISL_15775590
18	cat	Seoul-036	2021-06-04	B.1.1.7	20I (Alpha, V1)	GRY	Alpha variant	EPI_ISL_15727690
19	dog	Seoul-038	2021-06-06	B.1.619.1	20A	GH	–	EPI_ISL_15775594
20	cat	Seoul-046	2021-06-30	B.1.619.1	20A	GH	–	EPI_ISL_15775583
21	cat	Seoul-049	2021-07-02	AY.69	21I (Delta)	GK	Delta variant	EPI_ISL_15727691
22	cat	Seoul-050	2021-07-11	AY.69	21I (Delta)	GK	Delta variant	EPI_ISL_15775584
23	dog	Seoul-053	2021-07-13	B.1.619.1	20A	GH	–	EPI_ISL_15727698
24	dog	Seoul-054	2021-07-14	AY.69	21I (Delta)	GK	Delta variant	EPI_ISL_15727699
25	dog	Seoul-059	2021-07-20	AY.69	21I (Delta)	GK	Delta variant	EPI_ISL_15727700
26	cat	Seoul-061	2021-07-22	AY.69	21I (Delta)	GK	Delta variant	EPI_ISL_15727692
27	dog	Seoul-065	2021-07-27	AY.69	21I (Delta)	GK	Delta variant	EPI_ISL_15793142
28	cat	Seoul-069	2021-08-01	B.1.619.1	20A	GH	–	EPI_ISL_15727693
29	dog	Seoul-071	2021-08-03	B.1.619.1	20A	GH	–	EPI_ISL_15727701
30	cat	Seoul-076	2021-08-08	AY.69	21I (Delta)	GK	Delta variant	EPI_ISL_15775585
31	cat	Seoul-077	2021-08-08	AY.69	21I (Delta)	GK	Delta variant	EPI_ISL_15727694
32	dog	07-Gyeonggi-North-01	2021-08-10	AY.69	21I (Delta)	GK	Delta variant	EPI_ISL_15775591
33	dog	Seoul-079	2021-08-10	AY.69	21I (Delta)	GK	Delta variant	EPI_ISL_15775595
34	dog	Seoul-081	2021-08-20	AY.69	21I (Delta)	GK	Delta variant	EPI_ISL_15727702
35	cat	Seoul-082	2021-08-24	AY.69	21I (Delta)	GK	Delta variant	EPI_ISL_15727695
36	cat	Seoul-093	2021-09-19	AY.69	21I (Delta)	GK	Delta variant	EPI_ISL_15775586
37	cat	Seoul-097	2021-10-08	AY.69	21I (Delta)	GK	Delta variant	EPI_ISL_15775587
38	cat	Seoul-098	2021-10-08	AY.69	21I (Delta)	GK	Delta variant	EPI_ISL_15775588
39	cat	Seoul-099	2021-10-24	AY.122.5	21J (Delta)	GK	Delta variant	EPI_ISL_15775589
40	dog	08-Gyeongnam-01	2021-12-10	AY.122.5	21J (Delta)	GK	Delta variant	EPI_ISL_15727697

† Nextstrain clade was determined by Nextclade (v2.8.1) (https://clade.nextstrain.org). ‡ GISAID Clade was determined based on the “Clade and lineage nomenclature, 2 March 2021” [10]

## Data Availability

All data are downloaded from GISAID (https://gisaid.org).

## Supplementary Materials

The following supporting information can be downloaded at: https://www.mdpi.com/article/10.3390/vetsci10010006/s1, Table S1: SARS-CoV-2 positive animals which samples were provided to APQA from Provincial/City government veterinary authorities for variants monitoring; Table S2: Results of Illumina sequencing of SARS-CoV-2 in dogs and cats; Table S3: 420 Korean human SARS-CoV-2 sequences used in the phylogenetic analysis.
