# Supplementary material for: Whole Genome Sequencing of SARS-CoV-2 in Cats and Dogs in South Korea in 2021
Source: Vet Sci. 2022 Dec 23;10(1):6. doi: 10.3390/vetsci10010006 (PMC9866686; doi:10.3390/vetsci10010006)
Supplement: Supplementary file 1 [file vetsci-10-00006-s001.zip › Supplementary_Tables S1 and S2.pdf]

Supplementary Table S1. SARS-CoV-2 positive animals which samples were provided to APQA from Provincial/City government veterinary authorities for variants monitoring

Province/City	Cats	Dogs	Total
Seoul	38	65	102
Daegu	-	1	1
Gwangju	1	-	1
Sejong	1	-	1
Gyeonggi	0	2	2
Gyeongnam	1	0	1
Jeonbuk	-	1	1
	41	69	110

Supplementary Table S2. Results of Illumina sequencing of SARS-CoV-2 in dogs and cats

No.	Species	Strain	# mapped reads	% mapped reads *	Average length (bp)	Total nucleotide sequences	Genome size (bp)	Coverage (x)
1	cat	01-Seoul-01	5,086,456	96.5	115	586,829,082	29,896	2,193.8
2	cat	02-Sejong-01	4,022,096	98.3	123	497,461,572	29,902	1,825.7
3	dog	Seoul-002	807,176	89.7	150	121,389,554	29,905	481.7
4	dog	03-Gyeonggi-01	1,922,464	96.1	127	245,540,138	29,896	1,464.4
5	cat	04-Gwangju-01	3,857,452	97.7	122	474,319,250	29,874	2,302.7
6	cat	Seoul-004	1,034,282	93.1	150	155,578,014	29,906	547.3
7	cat	Seoul-005	4,257,408	98.5	125	532,586,970	29,903	2,474.3
8	cat	Seoul-006	4,188,844	98.2	125	524834,426	29,903	2,400.2
9	cat	Seoul-007	4,625,324	98.3	125	581,826,502	29,903	2,497.9
10	cat	Seoul-014	2,705,296	98.2	127	345,727,954	29,891	1,978.6
11	dog	05-Daegu-01	4,890,204	98.3	124	608,292,524	29,903	2,537.6
12	dog	Seoul-016	2,425,346	98.0	128	311,669,414	29,874	1,842.2
13	cat	Seoul-018	2,208,780	98.5	128	284,289,108	29,894	1,734.3
14	cat	Seoul-19	7,785,030	98.1	122	950,116,286	29,893	2,559.0
15	cat	Seoul-28	2,751,768	97.6	126	349,218,456	29,903	1,956.6
16	cat	Seoul-29	3,703,022	97.9	125	463,667,784	29,903	1,816.2
17	dog	06-Jeonbuk-01	4,683,960	92.7	115	540,160,400	29,871	2,594.3
18	cat	Seoul-036	4,127,046	98.6	126	523,068,192	29,883	2,386.6
19	dog	Seoul-038	3,801,266	97.5	121	462,279,280	29,869	2,248.0
20	cat	Seoul-046	3,321,530	97.7	123	411,047,684	29,886	1,784.1
21	cat	Seoul-049	4,112,840	98.3	124	510,836,652	29,890	2,475.1
22	cat	Seoul-050	3,876,810	98.5	126	490,541,102	29,890	2,330.2
23	dog	Seoul-053	4,896,398	98.3	124	607,393,692	29,893	2,615.4
24	dog	Seoul-054	6,489,812	98.1	121	790,647,596	29,890	2,359.5
25	dog	Seoul-059	2,151,876	98.0	127	273,860,334	29,890	1,755.3
26	cat	Seoul-061	2,248,074	98.0	127	287,740,460	29,890	1,629.0
27	dog	Seoul-065	5,313,056	97.6	121	648,118,512	29,884	2,675.1
28	cat	Seoul-069	3,666,434	98.0	122	448,043,920	29,893	2,199.2
29	dog	Seoul-071	4,073,256	97.8	125	513,108,526	29,893	2,453.9

30	cat	Seoul-076	3,482,942	96.7	91	319,058,188	29,891	2440.0
31	cat	Seoul-077	2,719,214	98.4	128	350,049,590	29,890	1,898.3
32	dog	07-Gyeonggi-North-01	2,834,718	81.9	120	342,888,082	29,877	1,875.1
33	dog	Seoul-079	3,993,942	95.9	124	498,228,148	29,895	2,226.6
34	dog	Seoul-081	4,376,248	97.6	127	557,002,376	29,890	2,449.8
35	cat	Seoul-082	2,993,146	97.2	126	377,302,156	29,890	2,009.6
36	cat	Seoul-093	3,780,048	98.3	126	479,637,102	29,889	2,322.9
37	cat	Seoul-097	4,418,244	98.2	126	557,507,462	29,889	2,480.9
38	cat	Seoul-098	5,621,860	97.7	123	695,358,720	29,885	2,725.7
39	cat	Seoul-099	4,413,264	97.9	126	557,565,960	29,888	2,395.7
40	dog	08-Gyeongnam-01	5,334,494	98.1	123	659,661,942	29,878	2,127.5

* The frequency of the lineage among positive samples in cats or dogs.
